# Calcium as a reliable marker for the quantitative assessment of endoplasmic reticulum stress in live cells

**DOI:** 10.1016/j.jbc.2021.100779

**Published:** 2021-05-14

**Authors:** Paul F. Lebeau, Khrystyna Platko, Jae Hyun Byun, Richard C. Austin

**Affiliations:** Department of Medicine, Division of Nephrology, McMaster University, The Research Institute of St Joe's Hamilton and Hamilton Centre for Kidney Research, Hamilton, Ontario, Canada

**Keywords:** calcium, endoplasmic reticulum stress (ER stress), unfolded protein response (UPR), Mag-Fluo-4, 4-PBA, 4-phenylbutyrate, Ca^2+^, calcium, CDN, CDN 1163, ER, endoplasmic reticulum, GRP, glucose-regulated protein, RyR, ryanodine receptor, SERCA, sarco/endoplasmic reticulum ATPase, TG, thapsigargin, TM, tunicamycin, TUDCA, tauroursodeoxycholic acid, UPR, unfolded protein response, VP, vasopressin

## Abstract

Calcium (Ca^2+^) is an essential mineral of endoplasmic reticulum (ER) luminal biochemistry because of the Ca^2+^ dependence of ER-resident chaperones charged with folding *de novo* proteins that transit this cellular compartment. ER Ca^2+^ depletion reduces the ability of chaperones to properly fold the proteins entering the ER, thus leading to an accumulation of misfolded proteins and the onset of a state known as ER stress. However, not all conditions that cause ER stress do so in a manner dependent on ER Ca^2+^ depletion. Agents such as tunicamycin inhibit the glycosylation of *de novo* polypeptides, a key step in the maturation process of newly synthesized proteins. Despite this established effect of tunicamycin, our understanding of how such conditions modulate ER Ca^2+^ levels is still limited. In the present study, we report that a variety of ER stress–inducing agents that have not been known to directly alter ER Ca^2+^ homeostasis can also cause a marked reduction in ER Ca^2+^ levels. Consistent with these observations, protecting against ER stress using small chemical chaperones, such as 4-phenylbutyrate and tauroursodeoxycholic acid, also attenuated ER Ca^2+^ depletion caused by these agents. We also describe a novel high-throughput and low-cost assay for the rapid quantification of ER stress using ER Ca^2+^ levels as a surrogate marker. This report builds on our understanding of ER Ca^2+^ levels in the context of ER stress and also provides the scientific community with a new, reliable tool to study this important cellular process *in vitro*.

The endoplasmic reticulum (ER) is a crucial organelle in the majority of cell types, known to play a key role in calcium (Ca^2+^) storage, lipid synthesis, detoxification of chemicals, and *de novo* peptide synthesis and maturation (1). Charged with such diverse tasks are a vast array of ER-luminal molecular chaperones, which rely on the highly specialized and regulated environment provided to them by the ER. Disturbances in this environment reduce chaperone activity, and thus, misfolded *de novo* polypeptides, lipids, and/or other toxins begin to accumulate in the ER lumen. This imbalance is known as ER stress and is an established driver of a variety of human diseases including neurodegenerative, cardiovascular, liver, kidney, and several metabolic diseases (2, 3).

Initiation of the unfolded protein response (UPR) resolves ER stress by inducing chaperone expression, thereby increasing the protein folding capacity of the ER (4). The UPR consists of a highly conserved signaling cascade orchestrated by three interrelated components: (a) the inositol-requiring enzyme 1α, (b) the activating transcription factor 6, and (c) the protein kinase R-like ER kinase. If the UPR fails to overcome this form of cellular stress, a proapoptotic program is initiated to remove the damaged cells from otherwise healthy tissue (5).

Ca^2+^ homeostasis is of critical importance for cells because of its role in regulating a variety of cellular processes including metabolism, phosphorylation, cell proliferation, division and differentiation, gene transcription, cell motility, muscle excitation-contraction, programmed cell death, and neurotransmission (6). ER luminal Ca^2+^ represents a fundamental component of the environment on which the majority of ER-resident chaperones depend (7, 8). To maintain elevated Ca^2+^ levels relative to the cytosol, three different classes of proteins must be expressed in the ER. These include (a) ER-resident Ca^2+^-binding proteins that increase the Ca^2+^-retaining ability of the ER, (b) ATP-dependent pumps, such as the sarco/endoplasmic reticulum ATPase (SERCA) for pumping Ca^2+^ into the ER lumen against the electrochemical gradient, and (c) Ca^2+^ channels, such as the ryanodine receptor (RyR) and inositol-trisphosphate receptor, which slowly release Ca^2+^ back into the cytosol for signaling purposes (9). It is well established that ER Ca^2+^ depletion caused by SERCA pump inhibitors, such as thapsigargin (TG), induces a robust UPR activation (10, 11). In a reciprocal manner, recent evidence also demonstrates that inhibition of channels or promoting SERCA pump activation can also protect against ER stress (12, 13). Not all conditions leading to ER stress, however, function directly through Ca^2+^-dependent mechanisms. Agents such as tunicamycin (TM) are known to inhibit N-glycosylation of newly synthesized proteins in the ER, a key step in the maturation of most secretory and cell-surface proteins, thus leading to their accumulation in this compartment (14). Furthermore, heritable loss-of-function protein mutants that transit the ER, such as vasopressin (VP) and α1-antitrypsin, fail to pass ER quality control and accumulate in the ER leading to ER stress and disease (15, 16). The effect of such conditions on ER Ca^2+^ levels, the fundamental regulator of ER chaperone function, is often overlooked and underappreciated.

In this report, we provide novel evidence that ER Ca^2+^ depletion not only promotes ER stress but can also occur as a result of ER stress. Although it is well established that agents such as TG significantly reduce ER Ca^2+^ levels, to our surprise, we also observed a significant reduction of ER Ca^2+^ upon treatment with ER stress–inducing agents that are not known to impact ER Ca^2+^ levels directly. Further confirmation and characterization of this unexpected occurrence also demonstrated that small chemical chaperones such as 4-phenylbutyrate (4-PBA) and tauroursodeoxycholic acid (TUDCA) attenuated ER Ca^2+^ loss resulting from TG- and TM-induced ER stress. Based on the reliability of ER Ca^2+^ levels to reflect the state of ER stress, we also describe a cost- and time-effective assay that takes advantage of the high-throughput and sensitive nature of fluorescent probes to use ER Ca^2+^ as a surrogate marker for the accurate quantitative assessment of ER stress in live cells.

## Results

### ER stress–induced changes in ER Ca^2+^ levels are readily detected with the low-affinity Ca^2+^ dye, Mag-Fluo-4 AM

**Figure 1:**
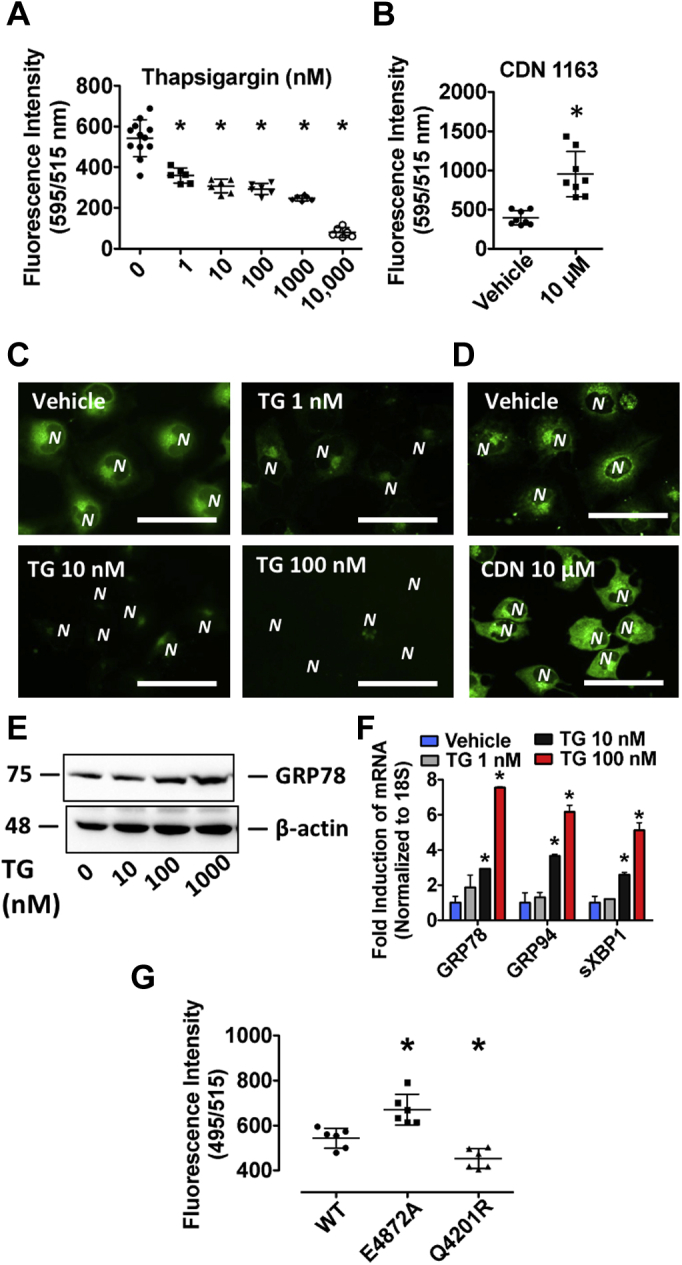
**Mag-Fluo-4 serves as a reliable tool for the detection of changes in ER Ca**^**2+**^**levels in live cells using a 96-well microtiter plate.***A* and *B*, ER Ca^2+^ levels were assessed in TG- and CDN-treated HuH7 cells loaded with Mag-Fluo-4 AM using a fluorescent spectrophotometer. *C* and *D*, the effect of TG and CDN on Mag-Fluo-4 AM fluorescence intensity was also visualized using a fluorescent microscope. *E* and *F*, increased ER stress marker expression in response to TG was confirmed in HuH7 cells via immunoblots and quantitative real-time PCR. *G*, ER Ca^2+^ levels were also examined in HuH7 cells transfected with the loss-of-function RyR2 variant, E4872A, and the gain-of-function RyR2 variant, Q4201R. ∗*p* < 0.05 *versus* vehicle-treated; scale bars represent 10 μm, and *N* denotes the position of a nucleus. Ca^2+^, calcium; CDN, CDN 1163; ER, endoplasmic reticulum; RyR2, ryanodine receptor 2; TG, thapsigargin.

In contrast to high-affinity indicators used to measure Ca^2+^ concentration in conditions of low abundance, such as the cytosol, low-affinity dyes can be used for the assessment of Ca^2+^ in cellular compartments with elevated Ca^2+^ levels, including the ER (17). Our need to understand and quantify the effect of ER stress on ER Ca^2+^ levels in a reliable and high-throughput manner led us to develop a low-cost microtiter plate assay using the Ca^2+^ dye, Mag-Fluo-4 AM. To validate the performance of the assay, a number of experiments were first carried out with established modulators of ER Ca^2+^. Consistent with previous studies (11), we observed that TG caused a marked reduction in ER Ca^2+^ levels, as indicated by a reduction in fluorescence intensity in HuH7 immortalized hepatocytes loaded with Mag-Fluo-4 AM (Fig. 1*A*). In contrast to these results, we observed that an agent known to increase SERCA pump activity, CDN 1163 (CDN) (13), increased fluorescence intensity in Mag-Fluo-4–loaded cells (Fig. 1*B*), indicative of an increase in ER Ca^2+^. Changes in fluorescence intensity of the ER using Mag-Fluo-4 AM were also visualized using a fluorescent microscope and were consistent with values obtained from the quantitative assay (Fig. 1, *C* and *D*). TG-induced ER stress was confirmed via immunoblot and real-time quantitative PCR analysis of ER stress markers including the glucose-regulated proteins of 78 kDa and 94 kDa (GRP78 and GRP94, respectively) and spliced X-box–binding protein. Genetically induced changes in ER Ca^2+^ levels were also assessed in HuH7 cells transfected with RyR2 variants, E4872A and Q4201R. Consistent with previous reports (18, 19), data generated using Mag-Fluo-4 AM suggest that the loss-of-function variant E4872A-RyR2 prevents ER Ca^2+^ leakage, whereas the gain-of-function Q4201R increases ER Ca^2+^ leakage (Fig. 1*G*).

### TM causes ER Ca^2+^ depletion in a variety of cultured cell lines 24 h after treatment

**Figure 2:**
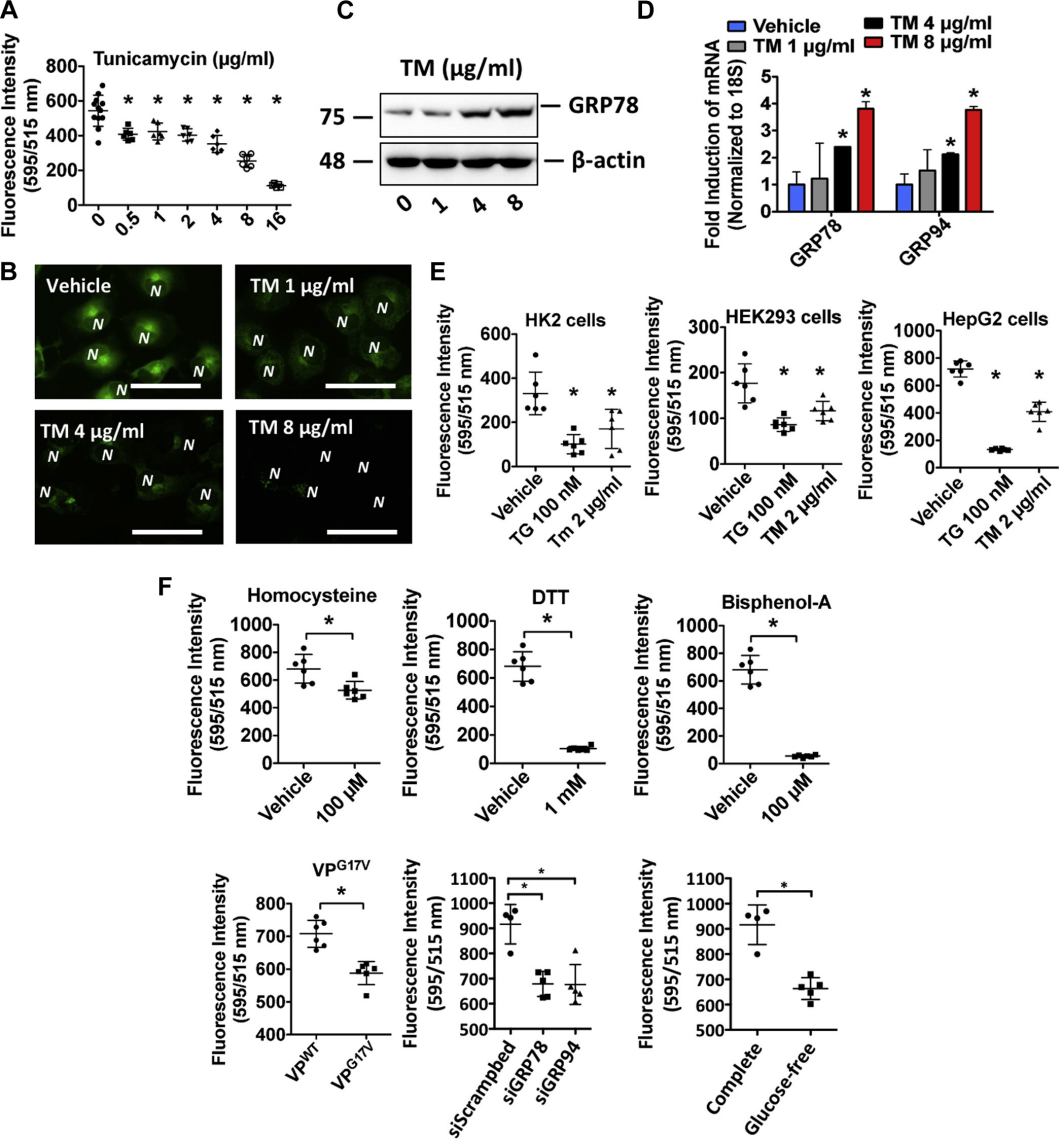
**A variety of conditions known to cause ER stress induce ER Ca**^**2+**^**depletion.***A* and *B*, HuH7 cells in a 96-well microtiter plate were treated with increasing concentrations of TM, loaded with Mag-Fluo-4 AM, and assessed for ER Ca^2+^ levels using a spectrophotometer and a fluorescent microscope. *C* and *D*, ER stress marker expression was examined in TM-treated HuH7 cells via immunoblots and real-time PCR. *E*, TG- and TM-induced changes in ER Ca^2+^ levels were also examined in immortalized kidney cells lines HK2 and HEK293, as well as in HepG2 immortalized hepatocytes. *F*, ER Ca^2+^ depletion was also examined in HuH7 cells treated with other well-established ER stress-inducing agents, homocysteine, DTT, and bisphenol-A as well as in cells transfected with a plasmid-encoding VP variant known to cause ER stress. ER Ca^2+^ depletion was also examined in Huh7 cells transfected with siRNA targeted against GRP78 and GRP94, as well as cells cultured in glucose-free medium. ∗*p* < 0.05 *versus* vehicle-treated; scale bars represent 10 μm, and *N* denotes the position of a nucleus. Ca^2+^, calcium; ER, endoplasmic reticulum; GRP78/94TG, thapsigargin; TM, tunicamycin; VP, vasopressin.

The ability of agents that affect SERCA pump activity to cause changes in ER Ca^2+^ levels is well established (20). It is also widely accepted that these agents affect the UPR in a manner dependent on such changes. Having optimized the sensitivity of the Mag-Fluo-4–based assay using established ER Ca^2+^-modulating agents, our next aim was to assess changes in ER Ca^2+^ levels in response to other well-established ER stress–inducing agents that are not known to cause ER stress in a manner dependent on changes in ER Ca^2+^ levels. Strikingly, similar to TG treatment, a dose-dependent reduction of ER Ca^2+^ was also observed in HuH7 cells treated with TM 24 h after treatment (Fig. 2*A*). TM-induced changes in fluorescence intensity were also visualized using a fluorescent microscope (Fig. 2*B*). UPR activation in response to TM was confirmed via assessment of GRP78 and GRP94 using immunoblots and real-time PCR (Fig. 2, *C* and *D*). The effect of TG and TM on ER Ca^2+^ levels was also examined in HK2 and HEK293 immortalized kidney cell lines as well as HepG2 human hepatocytes subjected to the same experimental conditions (Fig. 2*E*).

Although TG and TM are among the most commonly used agents to study the impact of ER stress in laboratory models, a variety of chemicals are known to negatively impact the ER environment and cause ER stress. Homocysteine has been shown to contribute to dyslipidemia via pathological activation of the sterol regulatory element-binding proteins, thus contributing to cardiovascular disease (21); DTT is a reducing agent capable of preventing disulfide bond formation of nascent proteins in the ER by chaperones such as protein disulfide isomerase and ERp72 (22); and bisphenol-A is an estrogen mimetic commonly found in plastics, capable of antagonizing the SERCA pump in a manner similar to TG (23). In addition, the accumulation of misfolded protein variants that fail to pass ER quality controls is also a known driver of ER storage diseases (24). VP variants, for instance, contribute to a form of autosomal dominant diabetes insipidus in a manner dependent on ER stress (16). Similarly, changes in the expression or activity of ER-resident Ca^2+^-binding proteins can affect their ability to bind and maintain elevated ER Ca^2+^ levels relative to the cytosol (6, 25). Furthermore, a variety of physiological conditions lead to UPR activation (26, 27, 28). For instance, glucose deprivation induced ER stress and apoptosis in cultured cells (26). Interestingly, we observed that all of the aforementioned agents and conditions caused the depletion of ER Ca^2+^ 24 h after treatment in HuH7 cells (Fig. 2*F*). We also demonstrate that culturing cells in a Ca^2+^-deprived medium resulted in ER Ca^2+^ depletion and ER stress, as demonstrated by ER Ca^2+^ quantification and quantitative real-time PCR analysis, respectively (Fig. S1, *A* and *B*). In line with previous findings (25, 26), siRNA-mediated knockdown of GRP78 and GRP94, as well as glucose deprivation, led to activation of the UPR response, which was confirmed by quantitative real-time PCR of established ER stress markers, including activating transcription factor 6, inositol-requiring enzyme 1, and protein kinase R-like ER kinase (Fig. S1, *C* and *D*) (6, 25, 26). In addition, cell viability after the treatment with known cytotoxic agents, such as DTT and bisphenol-A, was also verified (Fig. S2, *A* and *B*).

**Figure 3:**
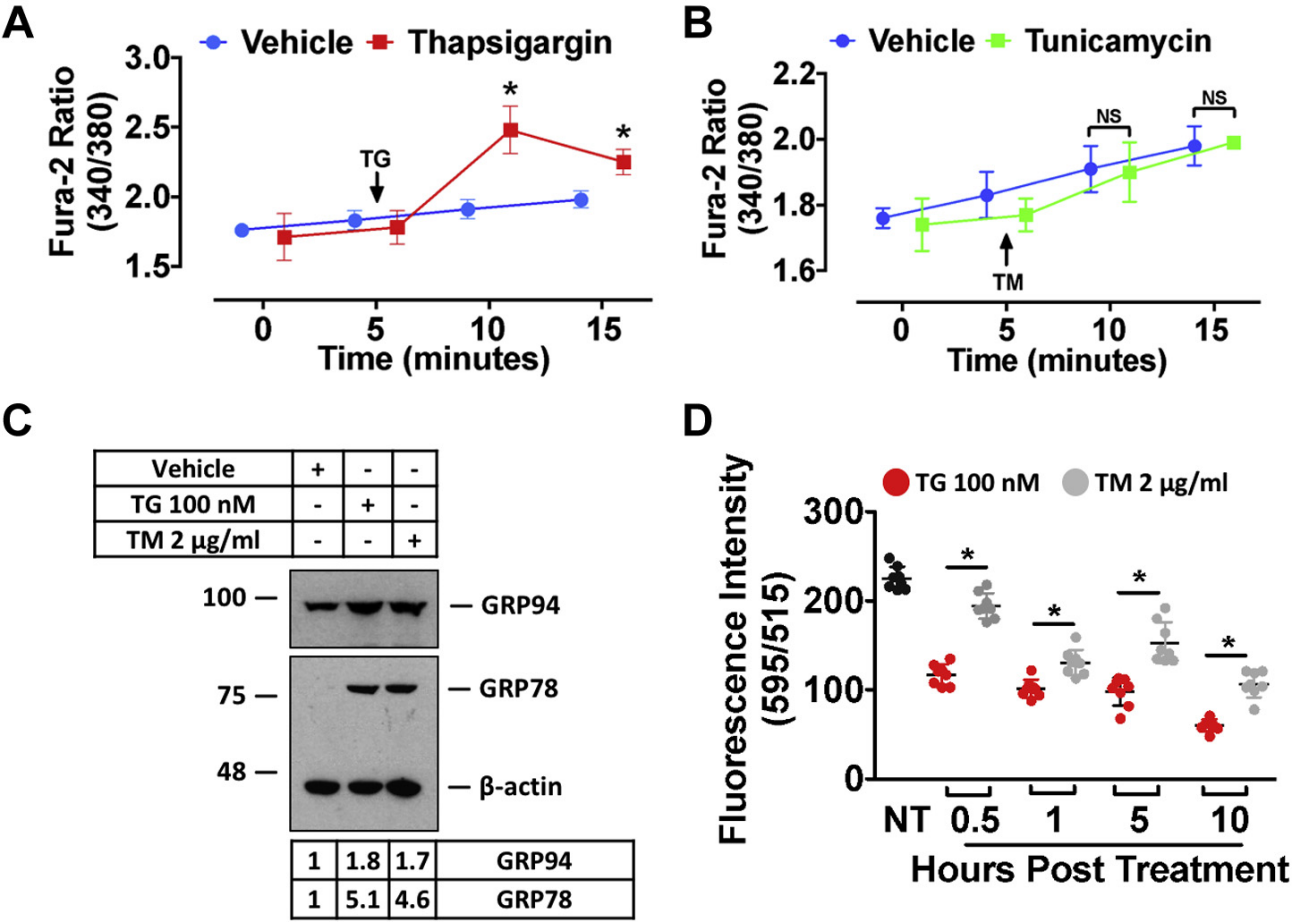
**TM does not affect ER Ca**^**2+**^**in a spontaneous manner like TG.***A* and *B*, Ca^2+^ release from the ER was examined in TG- (1 μM) and TM- (20 μg/ml) treated HuH7 cells using the high-affinity Ca^2+^ dye, Fura-2 AM. Each of the drugs was added to the cells immediately after the 5-min time point. *C* and *D*, the doses of TG and TM capable of causing similar induction of ER stress markers GRP78 and GRP94 were established via immunoblot. HuH7 cells were then treated with the indicated doses to allow for a direct comparison of the ER Ca^2+^-depleting abilities of these compounds. ∗*p* < 0.05 *versus* vehicle-treated. Ca^2+^, calcium; ER, endoplasmic reticulum; GRP, glucose-regulated protein; NS, nonsignificant; TG, thapsigargin; TM, tunicamycin.

To support our hypothesis that TM causes ER Ca^2+^ depletion as a secondary byproduct of ER stress, additional assays were carried out using a high-affinity cytosolic Ca^2+^ dye to assess its ability to spontaneously affect ER Ca^2+^ release. Consistent with previous reports (11), TG caused a spontaneous release of Ca^2+^ from the ER (Fig. 3*A*). In contrast, TM did not significantly impact the release of Ca^2+^ from the ER into the cytosol (Fig. 3*B*). A direct comparison of ER Ca^2+^ was then carried out in HuH7 cells treated with doses of TG and TM that caused a similar level of UPR activation 10 h after treatment, as indicated by GRP78 and GRP94 immunoblots (Fig. 3*C*). Consistent with previous experiments, TG and TM induced a reduction of ER Ca^2+^ levels compared with vehicle-treated cells. Importantly, however, TG reduced ER Ca^2+^ levels to a greater extent than TM at all time points examined (Fig. 3*D*). Given that TM does not cause a spontaneous release of Ca^2+^ from intracellular stores and fails to cause ER Ca^2+^ depletion to the same extent as TG, these data suggest that TM-induced changes in ER Ca^2+^ occur as a downstream result of ER stress.

### Small chemical chaperones protect against ER stress and attenuate ER Ca^2+^ depletion

**Figure 4:**
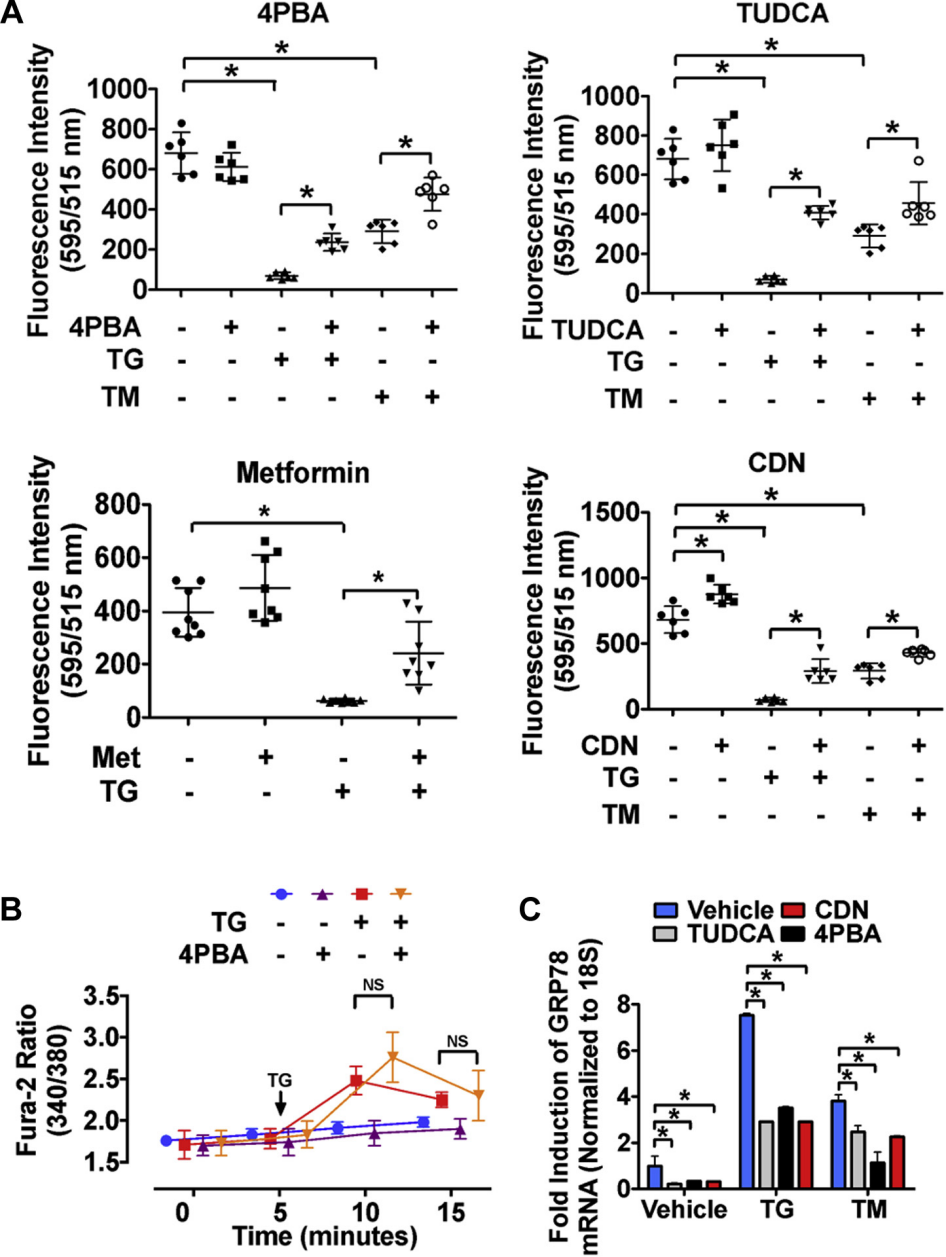
**Small chemical chaperones protect against ER stress and attenuate ER Ca**^**2+**^**loss.***A*, HuH7 cells seeded in a 96-well microtiter plate were pretreated with the small chemical chaperones 4-PBA (1 mM) or TUDCA (500 μM), as well as metformin (500 μM) or CDN (10 μM) for 24 h and then exposed to either TG (100 nM) or TM (2 μg/ml) for an additional 24 h. ER Ca^2+^ levels were then quantified using Mag-Fluo-4 AM. *B*, the effect of 4-PBA pretreatment on TG-induced ER Ca^2+^ depletion was also assessed in HuH7 cells using the cytosolic Ca^2+^ dye, Fura-2 AM. *C*, the ability of 4-PBA, TUDCA, and CDN to protect against ER stress was confirmed via real-time PCR analysis of ER stress marker, GRP78. ∗*p* < 0.05. Ca^2+^, calcium; CDN, CDN 1163; ER, endoplasmic reticulum; GRP, glucose-regulated protein; NS, nonsignificant; 4-PBA, 4-phenylbutyrate; TG, thapsigargin; TM, tunicamycin; TUDCA, tauroursodeoxycholic acid.

To further assess the role of ER stress as an upstream effector of ER Ca^2+^ levels, our next aim was to examine whether small chemical chaperones and other agents known to protect against ER stress (29, 30, 31) can also restore ER Ca^2+^ levels. Accordingly, HuH7 cells were pretreated with 4-PBA, TUDCA, metformin, or CDN for 24 h and then exposed to dimethyl sulfoxide vehicle, TG, or TM for an additional 24 h before the Mag-Fluo-4 assay (Fig. 4*A*). Results from these experiments demonstrate that (a) 4-PBA, TUDCA, and metformin treatment alone does not affect ER Ca^2+^ but that (b) these agents prevent ER Ca^2+^ loss during conditions of TG- and TM-induced ER stress. The SERCA pump activator, CDN (13), increased ER Ca^2+^ levels in the absence and in the presence of TG or TM. Interestingly, we also observed that 4-PBA did not attenuate the ability of TG to cause an influx of Ca^2+^ from the ER into the cytosol (Fig. 4*B*). Given that 4-PBA protects against ER stress (Fig. 4*C*) but does not directly affect ER Ca^2+^ transients, these data also support the notion that ER Ca^2+^ can be altered as a result of ER stress.

## Discussion

ER stress is a well-studied cellular stress pathway that can contribute to a number of disease states (2). However, there remains much to be uncovered about the intricacies of the UPR as well as the secondary messengers that modulate its signaling cascades. In this report, we demonstrate that ER stress–inducing agents such as TM fail to promote immediate changes in ER Ca^2+^ release but that prolonged 24-h exposure of TM can reduce ER Ca^2+^ levels to a similar extent as established Ca^2+^-modulating agents such as TG.

The homeostasis of ER Ca^2+^ is maintained by an elaborate system of ER-resident Ca^2+^ transporters, Ca^2+^ channels, and Ca^2+^-binding/buffering proteins. Any changes in expression or activity of these components can impact ER Ca^2+^ levels (6, 9). Given the number of variables that play a role in this equilibrium, the exact mechanisms by which the inhibition of N-glycosylation by TM, or other conditions that cause of ER stress, affect ER Ca^2+^ levels are likely numerous. For instance, SERCA-mediated Ca^2+^ uptake was increased in conditions of ER stress induced by ER Ca^2+^ depletion as well as TM (32). In another example, TM treatment induced the production of oxidative species in smooth muscle cells (33), while oxidation of RyR in the skeletal muscle was associated with persistent activation of RyR channels, which was accompanied by an increase in Ca^2+^ efflux from the ER (34). Similar to our findings, Yamamoto *et al.* (35) demonstrated that TM treatment induced ER Ca^2+^ release in β-pancreatic cells. Furthermore, TM directly induced activation of the RyR, whereas no change in RyR expression or inositol-trisphosphate receptor function was observed (35). Because of the protein-centric nature of ER-resident Ca^2+^ modulators, it is also possible that TM-mediated ER stress reduces the ability of the ER to produce or replace old/damaged pumps and/or channels at the ER membrane. In line with this notion, studies demonstrate that TM can induce protein misfolding within the ER (36) and subsequent UPR activation (11), which could have an independent effect on ER Ca^2+^ homeostasis. Collectively, these findings demonstrate that in addition to its effect on N-glycosylation and UPR activation, TM also indirectly modulates ER Ca^2+^ balance.

Protein chaperones found within the ER lumen are critical in maintaining ER Ca^2+^ homeostasis (6, 25). Chaperones such as GRP78 and GRP94 are especially important because they play a dual role as regulators of both the UPR and ER Ca^2+^ (25). Consistent with its role as an ER chaperone, GRP94 binds to nascent polypeptides in the ER lumen, a process that is stabilized by Ca^2+^ (37). In addition, to help maintain ER homeostasis, GRP94 prevents Ca^2+^ leakage from the ER lumen (38). ER stress–inducing compounds, such as TM (35) and TG (11), as well as physiologic conditions that induce ER stress, including hypoxia (27) and ischemia (28), have been shown to increase the expression of GRP94 and ER Ca^2+^. Furthermore, changes in the expression and activity of other Ca^2+^-binding chaperones, including protein disulfide isomerase and calreticulin, are known to bear significant repercussions on ER Ca^2+^ levels and ER function, as these proteins contribute to Ca^2+^ buffering and stabilize the interaction between polypeptides and other chaperones (25, 39). Thus, overburdening of ER chaperones with misfolded *de novo* protein and/or alterations in the glycosylation status of these chaperones may also contribute to a reduction in their ability to bind and maintain elevated ER Ca^2+^ levels relative to the cytosol (40).

Previous studies have identified the Ca^2+^ binding potential of many chaperones, including low-affinity/high-capacity binders (such as GRP94) and high-affinity binders (such as calreticulin) (41). In this study, our findings highlight a disconnect in which ER Ca^2+^ levels can become independent of ER chaperone expression, perhaps identifying a novel characteristic of the ER environment during conditions of ER stress. Importantly, previous studies have demonstrated that the ability of chaperones to bind ER Ca^2+^ can be dependent on the availability of other cochaperones or molecules, such as Mg^2+^ and ADP/ATP in the case of GRP78 (42). Although the expression of ER chaperones increases during conditions of ER stress, our findings as well as those of others suggest that the Ca^2+^-binding capacity of such chaperones is proportional to the overall health of the ER environment. Despite the growing attention given to ER stress by the scientific community, there remains a limited number of tools available for its assessment (43, 44). In this report, we describe a novel application for the low-affinity Ca^2+^ dye, Mag-Fluo-4 AM, to quantify ER stress in cultured cells. The Mag-Fluo-4 AM–mediated Ca^2+^ quantification provides an accurate, time- and cost-effective alternative for quantifying ER stress, which is also comparable to the conventional gold-standard molecular biology tools that are traditionally used to examine ER stress. In support of this notion, our data highlight ER Ca^2+^ as a highly sensitive marker of ER stress; immunoblots revealed changes in GRP78 expression in response to a 24-h treatment of TG and TM at doses of 100 nM and 4 μg/ml, respectively. In contrast, the fluorescence-based assay detected changes in ER Ca^2+^ levels at significantly lower doses of TG (1 nM) and TM (0.5 μg/ml). Thus, ER Ca^2+^ is a sensitive and reliable surrogate marker of ER stress that is also advantageous because of a high-throughput detection potential. This assay also demonstrates superiority over the traditional methods with reduced reagent and equipment requirements. Although the fluorescence-based method has many advantages over conventional techniques used to assess ER stress, it is not without limitations. One of the limitations of this method is the potential for background signal, known as autofluorescence, which can affect quantification. Alternatively, prolonged exposure of experimental samples to light can also result in photobleaching of the fluorescent probes and alter Ca^2+^ quantification. Among other factors that are known to influence fluorescence-based assays are pH and temperature (45). It is also important to note that this technique may not be suitable for all cell types because of the use of pluronic acid, which may cause cell detachment in certain types of cultured cells (46). Overall, this report builds on our knowledge of the ER environment, as well as the behavior of its chaperones, and also describes a new tool that molecular biologists can exploit for the high-throughput and sensitive quantification of ER stress–induced changes in ER Ca^2+^ levels *in vitro*.

## Experimental procedures

### Cell culture, transfections, and treatments

HuH7, HepG2, HK2, and HEK293 cells were routinely grown in complete Dulbecco’s modified Eagle's medium containing 10% fetal bovine serum, 100 IU/ml penicillin, and 100 μg/ml streptomycin (Gibco, Thermo Fisher Scientific). WT VP or the G17V mutant cloned into the pEGFP-N1 plasmid (16), as well as loss- and gain-of-function RyR2 variants, E4872A and Q4201R, were generous gifts from Drs. David Cool (Wright State University) and Wayne Chen (University of Calgary), respectively. For experiments aimed at examining the effect of the expression of mutant proteins on ER Ca^2+^, appropriate plasmids were transfected into HuH7 cells using the X-tremeGENE HP DNA (Sigma-Aldrich) transfection reagent as per manufacturer’s instructions. TG, TM, 4-PBA, TUDCA, DTT, and homocysteine were purchased from Sigma-Aldrich. All cell treatments using pharmacologic inducers of ER stress were performed over a course of 24 h unless stated otherwise.

### Development of a novel 96-well microtiter assay for detecting ER stress by quantifying ER Ca^2+^ levels

A quantitative method was developed for the high-throughput and sensitive detection of ER stress by means of quantifying ER Ca^2+^ levels using the low-affinity Ca^2+^ dye, Mag-Fluo-4 AM. Briefly, cells were seeded in a black clear-bottom 96-well plate and permitted to rest for 24 h. The following day, cells were treated with ER stress–inducing agents for an additional 24 h. The medium was then removed and cells washed with Hank’s Buffered Saline Solution containing 20 mM Hepes. Cells were then exposed to a solution containing 6 μM Mag-Fluo-4 AM (Thermo Fisher Scientific) and 2% pluronic acid (v/v; Thermo Fisher Scientific) for 1 h in the dark at 37 °C. Excess dye was then washed off using Hank’s Buffered Saline Solution, and fluorescence intensity in live cells was measured using a fluorescent spectrophotometer at a wavelength of 495ex/515em (Molecular Devices, Gemini EM). Assessment of cytosolic Ca^2+^ levels was carried out using the high-affinity ratiometric Ca^2+^ dye, Fura-2 AM (Thermo Fisher Scientific), as described previously (11).

### Immunoblotting

Cells were lysed in the SDS-PAGE lysis buffer and equally loaded into polyacrylamide gels and electrophoretically resolved before being transferred to nitrocellulose membranes. Membranes were blocked in a tris-buffered saline solution containing 5% skim milk. Membranes were then incubated in tris-buffered saline containing primary antibodies targeted against GRP78 (610979, BD Bioscience), GRP94 (ADI-SPA-850, Enzo Life Sciences) or β-actin (A228, Sigma-Aldrich) for 18 h at 4 °C. Excess primary antibodies were washed from the membranes, and protein abundance was visualized using horseradish peroxidase-conjugated secondary antibodies (Bio-Rad) and EZ-ECL chemiluminescent reagent (FroggaBio).

### Quantitative real-time PCR

RNA was isolated using RNeasy Mini Kits (Qiagen), reverse-transcribed to cDNA using SuperScript VILO cDNA synthesis kits (Thermo Fisher Scientific), and assessed using Fast SYBR Green Master Mix and the ViiA 7 real-time PCR system (Thermo Fisher Scientific) as described previously (47). For the quantitative real-time PCR, the following human primer forward (F) and reverse (R) sequences were used: GRP78, F: 5′-CATCACGCCGTCCTAT GTCG-3′, R: 5′-CGTCAAAGACCGTGTTCTC G-3′; GRP94, F: 5′-TACTATGCGAGTCAGAAGAAAACA-3′, R: 5′-CATTCTTTCTATTCTATCTCCA TA-3′; spliced X-box–binding protein, F: 5′-CCGCAGCAGGTGCA GG-3′, R: 5′-GAGTCAATACCGCCAGAA TCCA-3′; 18S, F: 5′- CAAGATCACCATCA CCAACG-3′, R: 5′-CAAGATCACCATCAC CAACG-3′.

### Statistical analysis

Data are represented as the mean and error bars as the SD. Statistical significance between two groups was calculated using unpaired Student’s *t*-tests, and comparisons between multiple groups was done using the one-way ANOVA with Tukey's multiple comparison testing. Differences were considered significant at *p* < 0.05.

## Data availability

All relevant data are presented in the main article and supporting information file.

## Supporting information

This article contains supporting information.

## Conflict of interest

The authors declare that they have no conflicts of interest with the contents of this article.
