# Supplementary material for: Calcium as a reliable marker for the quantitative assessment of endoplasmic reticulum stress in live cells
Source: J Biol Chem. 2021 May 14;296:100779. doi: 10.1016/j.jbc.2021.100779 (PMC8191341; doi:10.1016/j.jbc.2021.100779)
Supplement: Figures S1 and S2 [file mmc1.pdf]

SUPPORTING MATERIALS

Calcium as a reliable marker for the quantitative assessment of endoplasmic reticulum stress in live cells

Paul F. Lebeau[1,], Khrystyna Platko[1,*], Jae Hyun Byun[1] and Richard C. Austin[1†]*

[1]Department of Medicine, Division of Nephrology, McMaster University, The Research Institute of St. Joe's Hamilton and Hamilton Centre for Kidney Research, Hamilton, Ontario L8N 4A6

* Both authors contributed equally to this work

† **To whom correspondence should be addressed:** 50 Charlton Ave. E., Rm. T-3313, Hamilton, Ontario L8N 4A6, Canada. Tel.: 905-522-1155 (ext. 35175); Fax: 905-540-6589; E-mail: austinr@taari.ca.

Running title: Calcium is an indicator of ER stress

Key Words: Calcium, endoplasmic reticulum stress (ER stress), unfolded protein response (UPR), Mag-Fluo-4

Supporting Materials Figure Legends

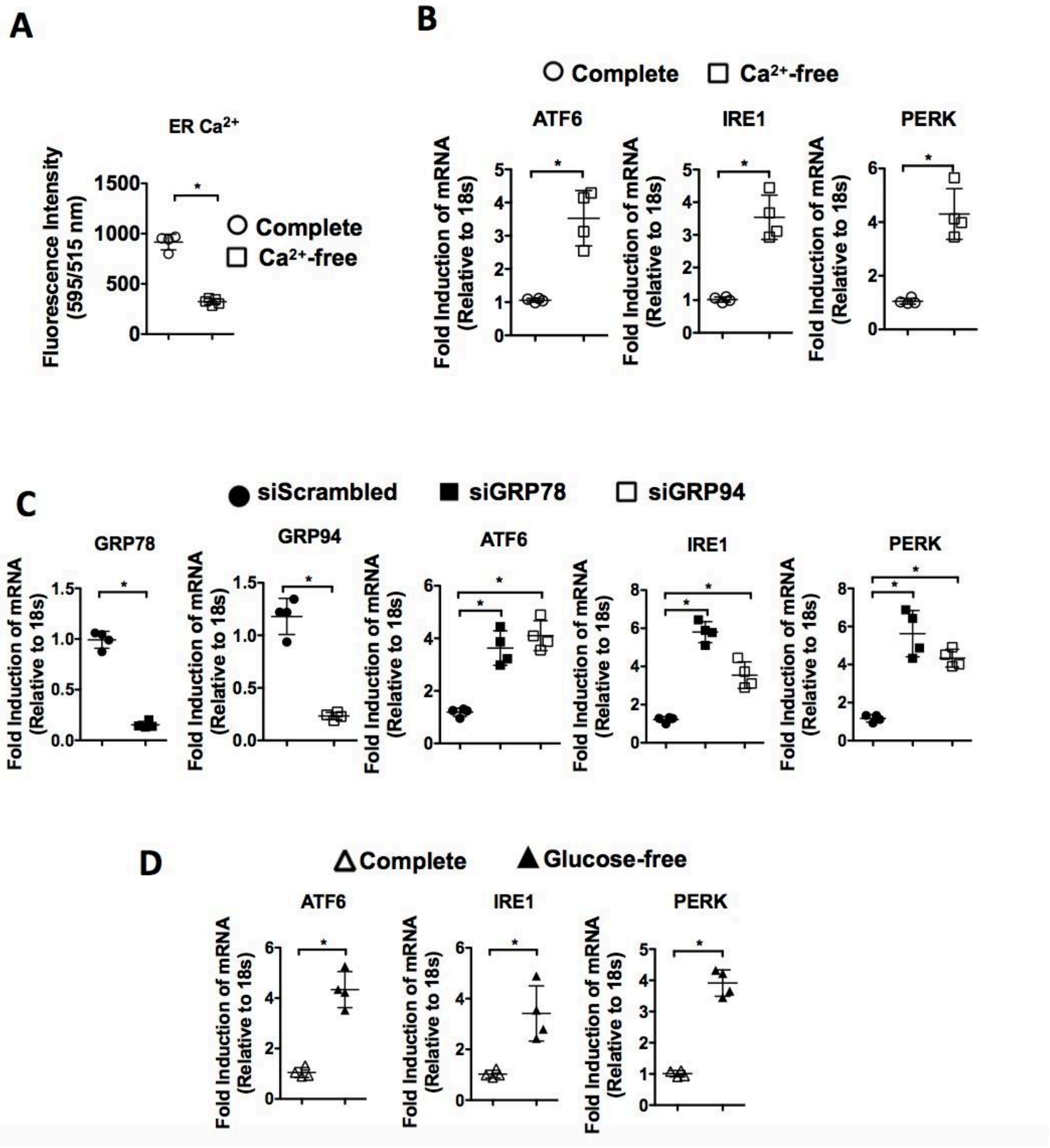

Fig. S1. A variety of conditions that lead to ER stress also cause ER Ca^{2+} depletion. (A) ER Ca^{2+} content and quantitative real-time PCR analysis of indicated genes in Huh7 cells cultured in Ca^{2+}-depleted medium for 48 hours. (B-D) Quantitative real-time PCR analysis of indicated genes. *, $p < 0.05$.

Figure S2

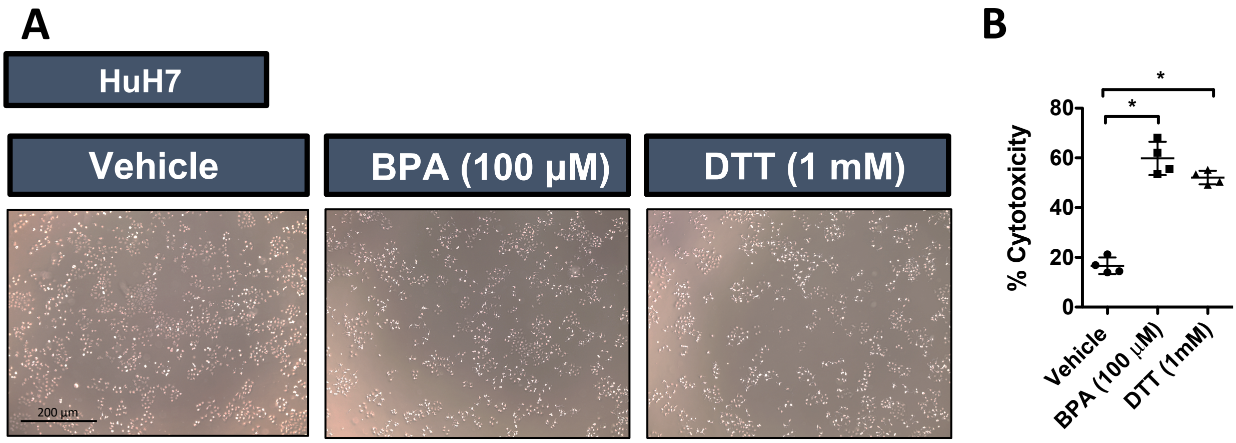

FigS2. Assessment of cell viability and cytotoxicity in Huh7 cells treated with BPA and DTT. (A) Morphological appearance of Huh7 cells treated with BPA (100 µM) and DTT (1 mM) for 48 hrs. (B) LDH- release quantification following a 24 hrs of BPA and DTT treatment in Huh7 cells. *, $p < 0.05$. BPA, bisphenol- A; DTT, dithiothreitol;
